# Designing a Cancer Prevention Collaborative Goal-Setting Mobile App for Non-Hispanic Black Primary Care Patients: An Iterative, Qualitative Patient-Led Process

**DOI:** 10.2196/28157

**Published:** 2022-03-24

**Authors:** Daniel Resnick, Matthew D Kearney, Jazmine M Smith, Allison Bautista, Liz Jones, Marilyn M Schapira, Jaya Aysola

**Affiliations:** 1 Department of Medicine Emory University School of Medicine Atalnta, GA United States; 2 Department of Family Medicine and Community Health Perelman School of Medicine University of Pennsylvania Philadelphia, PA United States; 3 Penn Medicine Center for Health Equity Advancement, Office of the Chief Medical Officer University of Pennsylvania Health System and Perelman School of Medicine University of Pennsylvania Philadelphia, PA United States; 4 Division of General Internal Medicine Perelman School of Medicine University of Pennsylvania Philadelphia, PA United States; 5 Transmogrify Conshohocken, PA United States; 6 Leonard Davis Institute of Health Economics University of Pennsylvania Philadelphia, PA United States; 7 The Center for Health Equity Research and Promotion Michael J Crescenz Veterans Administration Medical Center Philadelphia, PA United States; 8 Office of Inclusion and Diversity Perelman School of Medicine University of Pennsylvania Philadelphia, PA United States

**Keywords:** mHealth, cancer prevention, goal setting, social networks, health disparities, primary care, accessibility, development, feasibility, mobile phone

## Abstract

**Background:**

There remains a need to engage at-risk primary care populations in cancer prevention behaviors, yet primary care physicians often lack the time or resources to discuss these behaviors with their patients.

**Objective:**

The objective of this study is to evaluate the content, usability, and acceptability of a mobile app that leverages insights from goal-setting and social network literature to facilitate cancer prevention goal setting, tracking, and sharing between non-Hispanic Black primary care patients and their social ties.

**Methods:**

We recruited eligible non-Hispanic Black primary care patients (aged ≥18 years) from 2 practice sites in West Philadelphia, using nonprobabilistic purposive sampling. We conducted semistructured interviews with 5 to 7 participants over 3 weeks to solicit feedback on paper mock-ups of the app, iteratively adapting these mock-ups after each set of interviews. Thereafter, and informed by initial feedback, we created an electronic beta version of the app and sought acceptability and usability feedback from a different set of participants. Then, we conducted content analysis of all user responses to search for unifying themes on acceptability and usability of both the initial mock-ups and beta version of the app. We further assessed app usability using questions derived from the System Usability Scale.

**Results:**

A total of 33 non-Hispanic Black primary care patients participated in this study. The mean age was 49 (SD 13) years, and 26 (79%) out of 33 participants identified as female. Semistructured interviews revealed three primary generalizable insights from our target population: the framing of each goal and its relevance to cancer impacted the likelihood that the goal would be chosen, participants thought that sharing health goals with others facilitates health behaviors, and most participants found it motivating to see other users’ goal progress, while still collaborating with these users on their health goals. An overarching insight that permeated across each theme was the participants’ desire to customize and personalize the app. Usability testing revealed that 100% (33/33) of participants found the app easy to use, and 76% (25/33) of participants reported that they would like to use this app frequently.

**Conclusions:**

Cancer prevention in the modern era must include options that are accessible to all, but this does not mean that all options must be universal. This study’s iterative process led to the development of a cancer prevention mobile app that non-Hispanic Black primary care patients deemed usable and acceptable and yielded noteworthy insights about what intended end users value in setting and accomplishing health goals.

## Introduction

### Background

Increasing the adoption of health behaviors at a population level is essential if we are to significantly decrease the burden of preventable cancer and improve public health. In the United States, more than 600,000 people die of cancer each year [1]. Approximately 30% of these deaths are linked to poor diet, physical inactivity, and carrying too much weight, with another 30% due to tobacco use, comprising nearly two-thirds of US cancer deaths [2-5]. Furthermore, cancer disproportionately impacts non-Hispanic Black populations largely due to inequities stemming from structural racism [6-9]. Evidence suggests that current primary care services do not effectively engage all patients in cancer prevention [10-16]. Therefore, there remains a need for other potential interventions to address this gap, especially among those most at risk.

One strategy to increase cancer prevention health behaviors is goal setting [17,18]. Collaborative goal setting, a process whereby the provider and patient agree upon a health-related specific SMART (specific, measurable, achievable, realistic, and time-bound) goal and action plan [19], has been shown to modify behaviors by directing intention and building self-efficacy [20-22]. However, in primary care, we lack an approach to implement a strategy for collaborative goal setting. A second approach to increase cancer prevention health behaviors is to disseminate health behaviors and knowledge through social networks, which are known to influence behaviors related to cancer risk, such as obesity and smoking [23-26]. Experimental studies suggest that reinforcement from multiple social ties (ie, through a network) increases health behavior adoption more than social reinforcements from single ties [27]. Prior work also suggests that cancer prevention strategies involving some form of social support are more effective in changing behaviors in BIPOC (Black, Indigenous, and People of Color) populations as compared with non-Hispanic White people [28,29]. There is evidence that BIPOC populations have denser social networks, with more reliable and frequent activation of informal social support [12,14].

The objective of this study is to develop and evaluate the content, usability, and acceptability of an electronic decision support tool—ie, a mobile app—that leverages these insights to facilitate cancer prevention goal setting, tracking, and sharing between primary care patients and their social ties. We conducted a series of semistructured interviews to determine the optimal content and app features before piloting the prototype with our priority population: non-Hispanic Black primary care patients. Although non-Hispanic Black populations use health technology at greater rates than their White counterparts [30-32], they remain underrepresented in studies about health technology and health behaviors [33,34]. There is evidence that end user experiences may vary by background and culture with the need for culturally sensitive and effective design [35-37]. In addition, there is a call for evaluating public health interventions with messaging grounded in the understanding of the populations served and without White bias [38,39].

### Objective

Given that this app will center on facilitating cancer prevention behaviors in populations most at risk, we aimed to ensure the app is culturally attuned to and meets the needs of its targeted end user. We also aimed to test features, such as leveraging social ties, given the evidence that such features may work better among non-Hispanic Black populations [28,29]. Therefore, this study aims to evaluate the content, usability, and acceptability of a cancer prevention app designed with direct input from and specifically for a non-Hispanic Black primary care patient population.

## Methods

### Study Overview

**Figure 1:**
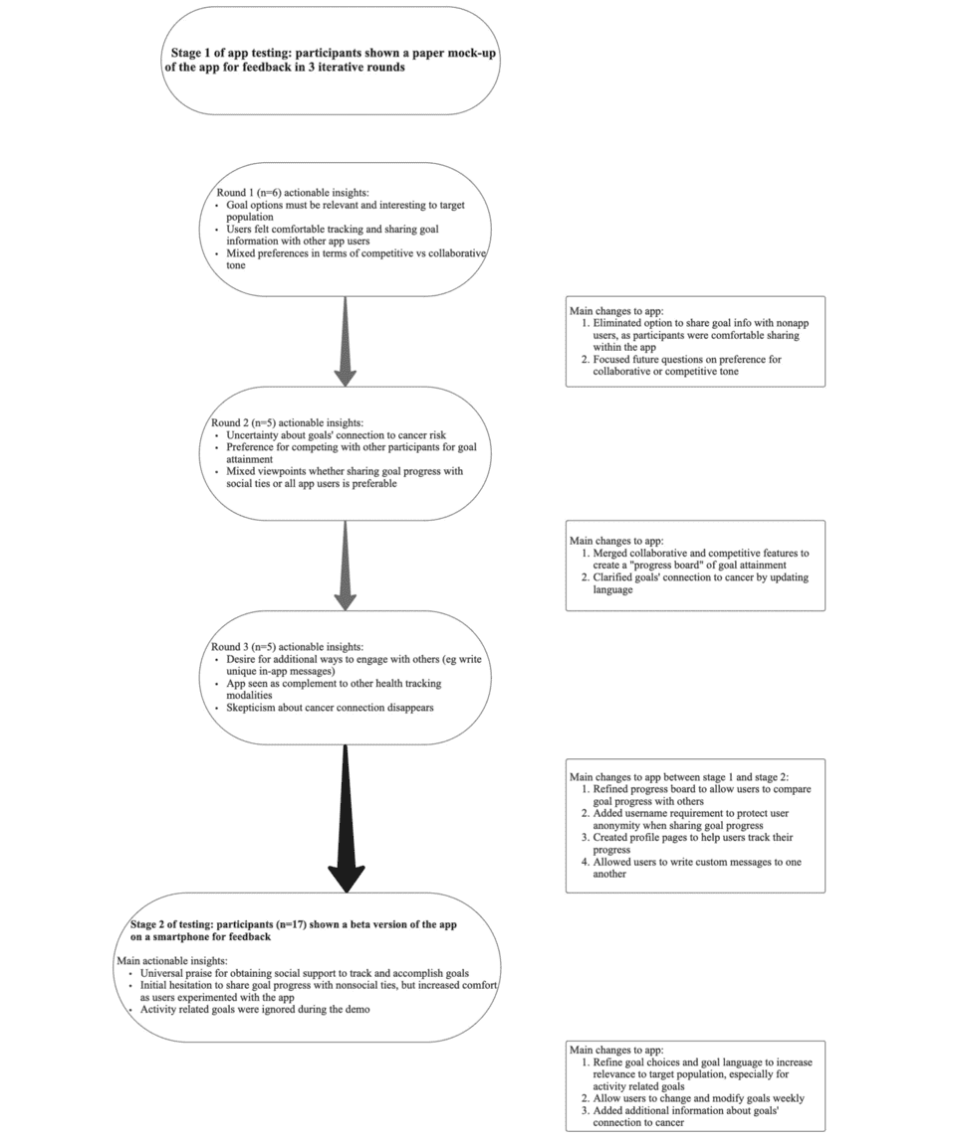
Visual representation of the app design process and the iterative changes made during app development.

We conducted a multistage, mixed methods study to develop and evaluate the content, usability, and acceptability of a mobile app that facilitates cancer prevention goal setting, goal tracking, and goal sharing. Consistent with mHealth app development best practices [40], this study comprised 2 stages. First, we obtained feedback from potential end users regarding the paper prototypes of the app. Then, we solicited feedback on a beta version of the app, informed by initial feedback, from a new set of participants. Participants completed questionnaires containing both open- and closed-ended questions, which were subsequently analyzed to refine the prototype. The study team guided the design, features, and content of both the paper prototype and the electronic beta version of the mobile app in collaboration with Transmogrify (Conshohocken, PA), a firm that helps create, build, and grow digital products. Figure 1 shows a visual representation of the study design and stages.

### Setting and Participants

#### Participants and Eligibility Criteria

Participants had to (1) self-identify as non-Hispanic Black, (2) be aged ≥18 years, (3) speak English, (4) be seen at one of our study sites once in the past 3 years (if the patient has a designated primary care provider [PCP]) or twice in the past 2 years (if no assigned PCP), (5) be able to provide informed consent, and (6) not have participated in another stage of testing for the intervention.

#### Setting and Recruitment Process

We recruited participants for both stages at 2 internal medicine primary care clinics in West Philadelphia, which serve a racially/ethnically diverse patient population, using a nonprobabilistic purposive sampling technique. We first generated a list of patients that met the first 4 eligibility criteria above with upcoming appointments. Then, between April and May 2019 (stage 1) and May and June 2019 (stage 2), study team members invited potential participants from the clinics’ waiting rooms that were on the pregenerated list to screen for the study. Interested participants reported to a private room after their appointments, where the study team confirmed the participants’ eligibility, including their self-reported race/ethnicity, informed participants of the study’s aims, and obtained formal consent for participation.

In stage 1, we aimed to recruit 5 to 7 participants per week over 3 weeks to rapidly modify the prototype based on participant feedback. Early usability testing research demonstrates that optimal feedback is derived from multiple rounds of testing with potential end users that inform refinements in between rounds [41-43], rather than one larger study that examines only one version of an app. In stage 2, we targeted a sample size of 15 to 20 participants to achieve thematic saturation of feedback and generate quantitative usability data of the beta version of the app [42,44]. Participants were incentivized at US $30 to complete the interviews. 

#### Ethics Approval

The University of Pennsylvania Institutional Review Board approved the protocol for this study (828151).

#### Mobile App Prototype

The main objectives of the mobile app prototype were to (1) communicate the value of collaborative goal setting for cancer prevention, (2) provide a selection of concrete SMART goals [45] related to cancer prevention behaviors informed by evidence-based guidelines and recommendations from the American Cancer Society (ACS) [46], and (3) serve as a patient-held prompt to facilitate cancer prevention collaborative goal-setting discussions with PCPs and encourage easy sharing of information with social ties. The earliest version of the prototype was based on guidelines for the adoption of cancer prevention behaviors [29]. We ensured it tested at a Flesch Reading Ease score of 86.2% (ie, is understood by 11- to 13-year-olds) and a Flesch-Kincaid grade level score of 4.3 (ie, is at a fourth-grade reading level) [47]. Through quantitative and qualitative assessments, we aimed to solicit end user perspectives and preferences on the apps’ (1) content and format, (2) delivery and use during primary care visits, and (3) use to share information with social ties (please refer to Multimedia Appendices 1 and 2 for iterative versions of our prototype at different stages of our study).

### Data Collection, Measurements, and Analysis

#### Qualitative and Quantitative Data Collection

##### Stage 1

Over a 3-week period, the study team conducted semistructured interviews with 5 to 7 participants each week to solicit feedback on paper mock-ups of the app. Semistructured interviews averaged 30 minutes and included both closed and open-ended questions about the app’s content, features, delivery methods, and potential future use. The study team synthesized and discussed the interview feedback weekly, iteratively refining the prototype before the subsequent set of participant interviews until the study team felt as though it could proceed to the next stage of testing. Please refer to Multimedia Appendix 1 for examples of our prototype and the corresponding interview guides.

##### Stage 2

Before stage 2, the development team transformed the latest paper prototype into an electronic beta version of the app. In stage 2, the study team walked individual participants (n=17) through the beta version of the app on a smartphone. During this 30-minute walk-through, team members asked each participant approximately 33 close-ended and approximately 20 open-ended questions about the usability and acceptability of the app and its features. Multimedia Appendix 2 illustrates an example of our prototype and interview guide.

For both stages, we recorded all open-and close-ended responses verbatim into REDCap (Research Electronic Data Capture; REDCap Consortium) [48].

#### Participant Characteristics

In stage 1, we assessed participants (n=16), age, sex, technology use, and health habits. Survey questions evaluated participants’ typical use of their mobile devices, comfortability with sharing health information with social ties on the internet and offline, and current goal setting and health tracking behaviors. In stage 2 (n=17), we collected the participants’ age and sex.

#### Qualitative Measures

We asked certain open-ended questions in both study stages to inquire about the participants’ overall impressions of the acceptability and usability of the app and its main features. We modified the interview guide iteratively each week in stage 1 to incorporate questions focused on specific changes made to the app based on the prior week’s feedback. Examples of our early prototypes and corresponding interview scripts are provided in Multimedia Appendix 1. The stage 1 prototype went through 10 refinements to inform stage 2. For stage 2, we developed this prototype within InVision [49], a digital product design platform that allows end users to interact with the prototype as if it is an app. Examples of this prototype and sample interview guides are provided in Multimedia Appendix 2.

#### Quantitative Measures

In addition to the open-ended questions, we asked participants in stage 2 (n=17) close-ended survey questions about the usability of certain app features and the app overall. These questions were adapted from the System Usability Scale [50], an instrument commonly used to evaluate the usability of different technology products.

#### Analysis

We first assessed the participants’ characteristics by tabulating the distributions or frequencies of the questions detailed above. We also calculated the distribution of Likert scale responses, ranging from strongly disagree to strongly agree, for the modified *System Usability Scale* questions asked in stage 2 of app testing.

#### Iterative Qualitative Analysis

Study team members (JMS, AB, LJ, and JA) met after each round of interviews to analyze feedback for key themes to inform refinements to the app content and features. This form of analysis allowed for the rapid implementation of the participants’ feedback and strengthened the development of the app [42].

#### Qualitative Content Analysis

Two team members (DR and MDK) conducted a qualitative content analysis [51] of all responses to search for unifying themes across stages of testing, by reading through the responses and creating the initial codebook. We randomly selected one interview from each round of testing (4 total) to refine the codebook and achieve consensus on code definition, inclusion criteria, and exclusion criteria. JMS then coded the remaining interviews using a constant-comparison technique. Throughout the coding process, a total of 8 out of 33 interviews (24% of the total sample) were jointly coded by DR, MDK, and JMS to assess interrater reliability. Facilitated by NVivo software (version 12; QSR International), we calculated the percent agreement [52], a measure of coding consensus, and determined that there was satisfactory interrater reliability (median 75% agreement; mean 69.4%, SD 23.5%). The study team then reviewed all coded responses and extracted key themes from across responses. This allowed for the simultaneous analysis of interviews collected throughout all stages of app development.

## Results

### Participant Characteristics

Of the 33 non-Hispanic Black primary care patients participating in the study, the mean age was 49 (SD 13) years, and 26 (79%) identified as female. Of the initial stage 1 (n=16) participants, 14 (88%) reported using a smartphone multiple times a day, 7 (44%) specifically used an app or digital fitness tracker to track their health, 13 (81%) reported tracking their health either digitally or manually, 15 (94%) reported sharing “some” or “a lot” of health information with close friends and family, and 13 (81%) said they have relied on friends to accomplish health goals. Only 25% (4/16) of the participants reported that they were comfortable discussing health matters on the internet.

### Qualitative Analysis

Table 1 summarizes the 3 dominant themes and associated subthemes that emerged from the qualitative content analysis of both stages. Below, we expand on these qualitative themes and add additional insights and changes made during the iterative analysis.

**Table 1:** Content themes and representative quotes.

Theme and subtheme		Representative quote
**Messaging matters**		
	SMART^a^ goals resonate	“It’s something that I’m already working on, so actually a lot of [the] options were pretty good, so I wanted to pick more than one of them” [stage 2]
	Achieving buy-in for cancer prevention messaging	“...[the app is] to the point. It tells me exactly what we’re working on and gives me some things right on hand to reduce my chances of getting cancer.” [stage 1, round 2]
	Specifying goals for the target population	“I was already interested in cutting down red meat, but I wasn't sure if I was ready to do it yet. So, it was cool to see that as an option.” [stage 2]
**To share or not to share**		
	Working with others facilitates goal accomplishment	“I like the idea of sharing with friends and family and seeing other people sharing their progress. Overall, I think it’s pretty good. It helps you keep on track.” [stage 2]
	Preferences for sharing goals with loved ones only versus all app users	“...I like [the app]. I would only pick [to share with] my friends. Since I'm trying to quit smoking, I wouldn't open it to everyone.” [stage 2]; “Family sometimes are critical. You could get more compassion from someone you don’t know.” [stage 1, round 2]
**Competition versus collaboration**		
	Deriving motivation from competition	“Ah yeah, some people like to do things out of competition.” Prompt: Would it be motivating for you personally? “Yes. I don’t like to lose.” [stage 1, round 2]
	Success through collaboration	“I think it’s a good idea to be able to communicate with [other users] the things that they are doing and the things that I'm doing to make better choices to reduce our risk of contracting cancer.” [stage 2]
	The progress board: a Goldilocks solution	“I really like the progress [board]. I like that you can click on a person and send them encouragement, or even your own personal message. I think I would use this app.” [stage 2]

^a^SMART: specific, measurable, achievable, realistic, and time-bound.

### Theme 1: Messaging Matters

#### SMART Goal Framing Resonates

A key objective of this app is to encourage the setting of appropriate and motivating health goals. Therefore, we sought feedback on how to present the SMART goals in a manner that is relevant to the target population, which highlights the important connection between these goals and cancer prevention. Overall, we found that messaging mattered to the participants. Participants appreciated the goals’ specificity and commented that these goals seem relevant to their efforts to become healthier. One participant stated as follows:

[The goals are] good...they’re all something that I can work on. I like the ‘Make small changes for a big impact.’ That makes a lot of sense.

stage 1, round 3

#### Achieving Buy-in for Cancer Prevention Messaging

A few participants noted confusion and skepticism about the relationship between lifestyle behaviors and cancer prevention. One participant said as follows:

only the smoking makes people think of cancer. The other ones seem more like basic health as opposed to associating it with cancer.

**Figure 2:**
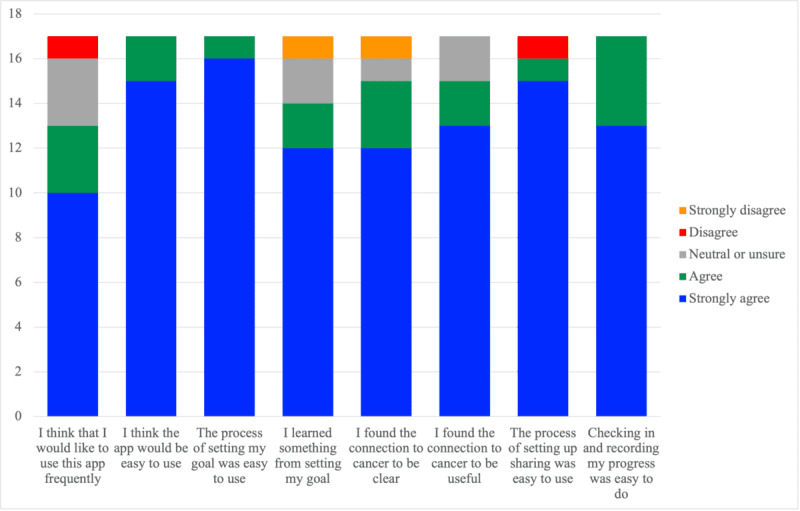
Usability feedback for key app features.

She then asked for “more specific information about how [these goals] relate to cancer.” [stage 1, round 2]. This initial feedback led to the following modifications: (1) we changed the language in the app to emphasize that small lifestyle modifications, such as those advocated by the app, can lead to a direct impact on cancer risk and (2) we added direct links to ACS webpages on lifestyle behaviors and cancer, to further emphasize the importance of these goals. Feedback on these modifications was positive in stage 2, with 15 (88%) participants out of 17 indicating that they agreed or strongly agreed that the connection to cancer in the app was both clear and useful. (See Figure 2 for additional quantitative results from stage 2).

#### Specifying Goals for the Target Population

In another example of the importance of SMART goal framing, we made significant changes to the app’s “Get Active” goals when we realized that none of the participants chose those goals when using the app in stage 2. Specifically, we centered each “Get Active” goal around a type of exercise (eg, “I will do 30 minutes of dancing”), rather than a more general goal (eg, “I will do 30 minutes of moderate intensity exercise”). As noted in a separate report, these new “Get Active” goals became more popular in future rounds of testing [53].

#### Desire for Customization

Participants broadly supported the goal choices offered by the app. However, nearly all participants wished to further adapt the goals themselves. Given this feedback, we added a number of customization options, from allowing users to choose the frequency of an action (eg, “I will do 30 minutes of dancing 4 times a week”) to permitting users to change their goals on a weekly basis. Even with these modifications, all goals remained SMART (ie, specific, measurable, achievable, realistic, and time-bound) and connected to cancer prevention.

### Theme 2: To Share or Not To Share

#### Working With Others Facilitates Goal Accomplishment

One key feature of this app is sharing health goals with other users and working on those goals together. Across the rounds of testing, participants universally agreed that this social component of the app was a valuable feature. One end user said as follows:

What I really like is the whole concept of sharing with someone else and getting them actively involved. It reminded me more of a safety plan [in the context of social work.] This is how we help you get where you need to get

stage 1, round 2

Participants expressed numerous benefits to working on health goals with others, from increased accountability for one’s own goals to the positive consequences of helping others.

#### Preferences for Sharing Goals With Loved Ones Only Versus All App Users

However, there is a lack of consensus about the user with whom health goals can be shared. Many users preferred only sharing their goal progress with the users they knew before joining the app. One participant did not want to reveal that he smokes outside his social circle, while another thought she would feel “pressured” by sharing her goals with all users. Nonetheless, some participants appreciated the opportunity to work on these health goals with all app users, with a couple of participants remarking that they may receive more valuable feedback from a user they did not know rather than from close friends and family. In addition, we noticed that as participants used the app in stage 2 of testing, their willingness to share the information with all app users increased. While using the app, 47% (8/17) participants selected “share with everyone.” In a postuse questionnaire, however, 82% (14/17) participants said they would select “share with everyone” in the future.

#### Customization Supports Personal Sharing Preferences

In response to different preferences, we modified the app to facilitate all aspects of goal sharing. Between stages 1 and 2, we added a username feature to protect anonymity when users shared goal information with unknown social ties on the app. We also permitted users to customize and change their share settings, allowing participants to choose whether to share information with only social ties or with all app users.

### Theme 3: Competition Versus Collaboration

#### Deriving Motivation From Competition

A third key feature of the app is helping users track their health goal progress, which participants universally agreed would facilitate goal accomplishment. Many participants also commented that it was motivating to view the progress of other users on the app. In fact, a number of participants suggested that the app should create a leaderboard to foster competition among users to rise to the top:

I definitely like the challenging [competitive version]. It’s good because it’s just like a game.

stage 1, round 2

#### Success Through Collaboration

Some participants strongly rejected the idea of a competitive tone on the app, with one woman stating as follows:

I’m not in competition with [other users] for my health...I don’t see where being in competition with someone else [is helpful]; there’s certain things I’m not competitive about and my health is one of them.

stage 1, round 3

Instead, many participants wanted the app to facilitate collaboration on health goals among users. Participants thought the app would be a valuable space to provide and receive suggestions on how to accomplish certain goals (eg, sharing recipes for healthy meals) and encouragement for goal progress.

#### The Progress Board: A “Goldilocks” Solution

In synthesizing this feedback on competition and collaboration, we changed the name of the “leaderboard” to “progress board,” so that users could visualize other users’ health goal progress without incentivizing competition. We also added a messaging feature because users expressed a strong interest in collaborating with one another. These changes resonated with participants in stage 2 of testing, with 88% (15/17) participants stating they would check their social ties’ goal progress weekly and 82% (14/17) stating they would send their friends encouraging messages weekly as well.

## Discussion

### Principal Findings

We conducted 2 stages of semistructured interviews with non-Hispanic Black primary care patients to develop and iteratively refine a cancer prevention goal setting mobile app. Our study yielded three primary generalizable insights from our target population: (1) the framing of each goal and its relevance to cancer impacted the likelihood that the goal would be chosen, (2) participants thought that sharing health goals with others facilitates the adoption of healthy behaviors, and (3) most participants found it motivating to see other users’ goal progress, while still collaborating with these users on their health goals. An overarching insight that emerged across themes was the participants’ desire to customize and personalize the app.

Our first theme highlights the importance of framing goals as relevant to cancer prevention. Some participants in our study initially struggled to understand the connection between cancer and lifestyle behaviors, remarking instead that they felt as though “genes” and “God’s will” had a larger role to play. Nonetheless, we maintained the overall cancer prevention framework of the app, as health behavior research has demonstrated that framing behaviors as cancer preventing increases their adoption [54-56]. Moreover, a 2019 meta-analysis found that underserved populations in the United States are comfortable receiving cancer prevention information and interventions on the internet and through mobile devices [57]. In response to the feedback received, we clarified the language on the app to better communicate how changes in behavior can make a difference in cancer risk. We re-enforced this message with links to the ACS lay resources. Following these changes, participants in subsequent rounds did not convey similar confusion, and in fact, many appreciated the link between health behavior and cancer prevention. This change is one example of the numerous adaptations we made to optimize the app for the intended end users.

The second and third themes indicate the values of goal sharing and collaboration, respectively. Although some participants hoped that the app would foster competition among users for completing health goals, most expressed a strong desire for the app to facilitate collaboration with others to achieve their health goals. This tension between competition and collaboration is frequently studied in behavioral economics, with mixed evidence as to which approach yields a greater impact on health behavior [58]. To respect the diverging opinions among our participants, we created a progress board that displays each user’s goal success count, allowing more competitive-minded users a chance to compare their progress to others. We removed any references to “leaderboards” and added new avenues for participants to communicate with each other on the app, responding to most participants who expressed a desire to collaborate with other app users. Brewer et al [59] similarly found a “sharing board” to be a popular feature in their app promoting cardiovascular health among church-going African Americans. Participants in Brewer’s study were likewise motivated by the ability to send and receive encouraging messages while keeping track of other users’ progress.

Finally, we found that participants uniformly valued the ability to customize the app to meet their unique needs, and in response, we provided additional options for goal sharing and goal setting. The desire for customization is common in health app development [60,61], and we aimed to satisfy participant requests while ensuring that the app remained grounded in evidence-based techniques. For example, although we allowed participants to modify the frequency of a particular health goal (eg, dance for 30 minutes three vs four times a week), we did not allow users to write their own health goals. We made this choice to ensure that all of our goals remained consistent with ACS recommendations and an evidence-based SMART [19] set-up.

Given that this study evaluated end user experience with a mobile app exclusively among a non-Hispanic Black population, it is difficult to ascertain whether our findings are unique to this population or perhaps more broadly applicable to other populations. However, we did find a preference for collaboration over competition that aligns with prior evidence of this among non-Hispanic Black populations [59]. In addition, our findings of fatalism in early iterations of app content feedback aligned with prior studies that demonstrate this among non-Hispanic Black populations [62-64]. Future studies should be designed to compare the experiences of apps and messaging between users to determine what features may be uniquely appreciated by one racial/ethnic population as compared with another.

### Strengths and Limitations

These findings must be considered in the context of several limitations and strengths. First, the study used a nonprobabilistic purposive sampling technique to recruit non-Hispanic black primary care patients from 2 primary care clinics and thus may not be generalizable. Second, we recognize that stated intentions do not always align with future behavior, and we cannot predict the effectiveness of this tool based on this study. The objective of this study is to develop and optimize the features and content of the app with and for our target population. This methodology also has several strengths. In contrast to many other behavior change apps [65], we followed a user-centered design approach to optimize the app for our intended audience, which may have different content wishes than other populations [57,66,67]. We also followed app design best practices by conducting iterative rounds of testing, allowing ample opportunities for usability feedback from potential end users [40,42]. Finally, 2 team members experienced in qualitative research analyzed all participant feedback using more traditional qualitative content analysis techniques to search for generalizable insights that may inform future health intervention research beyond the development of this app.

### Conclusions

Cancer prevention in the modern era must include options that are accessible to all, but this does not mean that all options must be universal. A mobile app—or any intervention, importantly—that promotes healthy, cancer-preventing behaviors in population A is not guaranteed to work as well with population B. Accordingly, given the disproportionate burden of cancer and cancer-related mortality among non-Hispanic Black populations in the United States [6-9], our iterative development approach for a cancer prevention mobile app focused uniquely and specifically on goal setting among non-Hispanic Black primary care patients. This iterative process led to the development of a cancer prevention mobile app that potential end users deemed usable and acceptable and yielded noteworthy insights about what intended end users value in health goals and how they may work on these goals with others.

## Appendix

Multimedia Appendix 1 Interview scripts and early prototype examples.

Multimedia Appendix 2 Interactive prototype and corresponding interview guide.

## Abbreviations

ACC: Abramson Cancer Center
ACS: American Cancer Society
BIPOC: Black, Indigenous, and People of Color
PCP: primary care provider
REDCap: Research Electronic Data Capture
SMART: specific, measurable, achievable, realistic, and time-bound

## References

[1] Siegel RL, Miller KD, Jemal A (2019). Cancer statistics, 2019. CA A Cancer J Clin.

[2] Katzke VA, Kaaks R, Kühn T (2015). Lifestyle and cancer risk. Cancer J.

[3] Lauby-Secretan B, Scoccianti C, Loomis D, Grosse Y, Bianchini F, Straif K, International Agency for Research on Cancer Handbook Working Group (2016). Body fatness and cancer--viewpoint of the IARC working group. N Engl J Med.

[4] Anand P, Kunnumakkara AB, Sundaram C, Harikumar KB, Tharakan ST, Lai OS, Sung B, Aggarwal BB (2008). Cancer is a preventable disease that requires major lifestyle changes. Pharm Res.

[5] Lortet-Tieulent J, Goding Sauer A, Siegel RL, Miller KD, Islami F, Fedewa SA, Jacobs EJ, Jemal A (2016). State-level cancer mortality attributable to cigarette smoking in the United States. JAMA Intern Med.

[6] Barbeau EM, Krieger N, Soobader MJ (2004). Working class matters: socioeconomic disadvantage, race/ethnicity, gender, and smoking in NHIS 2000. Am J Public Health.

[7] Krieger N (2005). Defining and investigating social disparities in cancer: critical issues. Cancer Causes Control.

[8] Kawachi I, Berkman LF, Berkman LF, Kawachi I, Glymour MM (2014). Social capital, social cohesion, and health Internet. Social epidemiology. 2nd edition.

[9] Berkman LF, Syme SL (1979). Social networks, host resistance, and mortality: a nine-year follow-up study of Alameda County residents. Am J Epidemiol.

[10] Aysola J, Bitton A, Zaslavsky AM, Ayanian JZ (2013). Quality and equity of primary care with patient-centered medical homes: results from a national survey. Med Care.

[11] Bach PB, Pham HH, Schrag D, Tate RC, Hargraves JL (2004). Primary care physicians who treat blacks and whites. N Engl J Med.

[12] Cornwell B, Schumm LP, Laumann EO, Graber J (2009). Social networks in the NSHAP study: rationale, measurement, and preliminary findings. J Gerontol B Psychol Sci Soc Sci.

[13] Freeman HP (2004). Poverty, culture, and social injustice: determinants of cancer disparities. CA Cancer J Clin.

[14] Haines VA, Hurlbert JS, Beggs JJ (1996). Exploring the determinants of support provision: provider characteristics, personal networks, community contexts, and support following life events. J Health Soc Behav.

[15] Katz SJ, Hofer TP (1994). Socioeconomic disparities in preventive care persist despite universal coverage. Breast and cervical cancer screening in Ontario and the United States. JAMA.

[16] Smedley BD, Stith AY, Nelson AR, Institute of Medicine (US) Committee on Understanding and Eliminating Racial and Ethnic Disparities in Health Care (2003). Unequal treatment: confronting racial and ethnic disparities in health care.

[17] Gotay CC (2005). Behavior and cancer prevention. J Clin Oncol.

[18] Strecher VJ, Seijts GH, Kok GJ, Latham GP, Glasgow R, DeVellis B, Meertens RM, Bulger DW (1995). Goal setting as a strategy for health behavior change. Health Educ Q.

[19] Bovend'Eerdt TJ, Botell RE, Wade DT (2009). Writing SMART rehabilitation goals and achieving goal attainment scaling: a practical guide. Clin Rehabil.

[20] Schunk DH (1990). Goal setting and self-efficacy during self-regulated learning. Educ Psychol.

[21] Heath C, Larrick RP, Wu G (1999). Goals as reference points. Cogn Psychol.

[22] Bandura A (1998). Health promotion from the perspective of social cognitive theory. Psychol Health.

[23] O'Malley AJ, Christakis NA (2011). Longitudinal analysis of large social networks: estimating the effect of health traits on changes in friendship ties. Stat Med.

[24] Centola D (2011). An experimental study of homophily in the adoption of health behavior. Science.

[25] Perkins JM, Subramanian SV, Christakis NA (2015). Social networks and health: a systematic review of sociocentric network studies in low- and middle-income countries. Soc Sci Med.

[26] Christakis NA, Fowler JH (2008). The collective dynamics of smoking in a large social network. N Engl J Med.

[27] Centola D (2010). The spread of behavior in an online social network experiment. Science.

[28] Wolff M, Bates T, Beck B, Young S, Ahmed SM, Maurana C (2003). Cancer prevention in underserved African American communities: barriers and effective strategies--a review of the literature. WMJ.

[29] Thomson CA, McCullough ML, Wertheim BC, Chlebowski RT, Martinez ME, Stefanick ML, Rohan TE, Manson JE, Tindle HA, Ockene J, Vitolins MZ, Wactawski-Wende J, Sarto GE, Lane DS, Neuhouser ML (2014). Nutrition and physical activity cancer prevention guidelines, cancer risk, and mortality in the women's health initiative. Cancer Prev Res (Phila).

[30] Krebs P, Duncan DT (2015). Health app use among US mobile phone owners: a national survey. JMIR Mhealth Uhealth.

[31] Chang E, Blondon K, Lyles CR, Jordan L, Ralston JD (2018). Racial/ethnic variation in devices used to access patient portals. Am J Manag Care.

[32] Ray R, Sewell AA, Gilbert KL, Roberts JD (2017). Missed opportunity? Leveraging mobile technology to reduce racial health disparities. J Health Polit Policy Law.

[33] Bennett GG, Steinberg DM, Stoute C, Lanpher M, Lane I, Askew S, Foley PB, Baskin ML (2014). Electronic health (eHealth) interventions for weight management among racial/ethnic minority adults: a systematic review. Obes Rev.

[34] James DC, Harville 2nd C, Sears C, Efunbumi O, Bondoc I (2017). Participation of African Americans in e-Health and m-Health studies: a systematic review. Telemed J E Health.

[35] AlJaberi H (2018). Developing culturally sensitive mHealth apps for Caribbean immigrant women to use during pregnancy: focus group study. JMIR Hum Factors.

[36] Alsswey AH, Al-Samarraie H, El-Qirem F, Alzahrani AI, Alfarraj O (2020). Culture in the design of mHealth UI: an effort to increase acceptance among culturally specific groups. Electron Libr.

[37] Joseph RP, Keller C, Vega-López S, Adams MA, English R, Hollingshead K, Hooker SP, Todd M, Gaesser GA, Ainsworth BE (2020). A culturally relevant smartphone-delivered physical activity intervention for African American women: development and initial usability tests of smart walk. JMIR Mhealth Uhealth.

[38] Vardeman-Winter J (2017). The framing of women and health disparities: a critical look at race, gender, and class from the perspectives of grassroots health communicators. Health Commun.

[39] Vardeman-Winter J (2011). Confronting whiteness in public relations campaigns and research with women. J Public Relat Res.

[40] Yardley L, Spring BJ, Riper H, Morrison LG, Crane DH, Curtis K, Merchant GC, Naughton F, Blandford A (2016). Understanding and promoting effective engagement with digital behavior change interventions. Am J Prev Med.

[41] Nielsen J, Landauer TK (1993). A mathematical model of the finding of usability problems. Proceedings of the INTERACT '93 and CHI '93 Conference on Human Factors in Computing Systems.

[42] Nielsen J (1994). Estimating the number of subjects needed for a thinking aloud test. Int J Hum Comput Stud.

[43] Virzi RA (1992). Refining the test phase of usability evaluation: how many subjects is enough?. Hum Factors.

[44] Mason M (2010). Sample size and saturation in PhD studies using qualitative interviews. Forum Qual.

[45] O'Neill J, Conzemius AE (2005). The power of SMART goals: using goals to improve student learning.

[46] (2020). American cancer society guidelines for nutrition and physical activity. American Cancer Society.

[47] Si L, Callan J (2001). A statistical model for scientific readability. Proceedings of the 10th International Conference on Information and knowledge management.

[48] Harris PA, Taylor R, Thielke R, Payne J, Gonzalez N, Conde JG (2009). Research electronic data capture (REDCap)– a metadata-driven methodology and workflow process for providing translational research informatics support. J Biomed Inform.

[49] InVision.

[50] Bangor A, Kortum PT, Miller JT (2008). An empirical evaluation of the system usability scale. Int J Hum-Comput Interact.

[51] White MD, Marsh EE (2006). Content analysis: a flexible methodology. Lib Trends.

[52] Stemler SE (2019). A comparison of consensus, consistency, and measurement approaches to estimating interrater reliability. Pract Assess Res Eval.

[53] Resnick D, Schapira MM, Smith JM, Bautista A, Xu C, Jones L, Aysola J (2021). Promoting collaborative goal setting for cancer prevention among primary care patients through mHealth: mixed methods evaluation of a new app. JMIR Form Res.

[54] Satia JA, Barlow J, Armstrong-Brown J, Watters JL (2010). Qualitative study to explore prospect theory and message framing and diet and cancer prevention-related issues among African American adolescents. Cancer Nurs.

[55] Webb TL, Sheeran P (2006). Does changing behavioral intentions engender behavior change? A meta-analysis of the experimental evidence. Psychol Bull.

[56] Gallagher KM, Updegraff JA (2012). Health message framing effects on attitudes, intentions, and behavior: a meta-analytic review. Ann Behav Med.

[57] Tarver WL, Haggstrom DA (2019). The use of cancer-specific patient-centered technologies among underserved populations in the United States: systematic review. J Med Internet Res.

[58] Patel MS, Small DS, Harrison JD, Fortunato MP, Oon AL, Rareshide CA, Reh G, Szwartz G, Guszcza J, Steier D, Kalra P, Hilbert V (2019). Effectiveness of behaviorally designed gamification interventions with social incentives for increasing physical activity among overweight and obese adults across the United States: the STEP UP randomized clinical trial. JAMA Intern Med.

[59] Brewer LC, Kumbamu A, Smith C, Jenkins S, Jones C, Hayes SN, Burke L, Cooper LA, Patten CA (2020). A cardiovascular health and wellness mobile health intervention among church-going African Americans: formative evaluation of the FAITH! app. JMIR Form Res.

[60] Coorey GM, Neubeck L, Mulley J, Redfern J (2018). Effectiveness, acceptability and usefulness of mobile applications for cardiovascular disease self-management: systematic review with meta-synthesis of quantitative and qualitative data. Eur J Prev Cardiol.

[61] Dugas M, Gao GG, Agarwal R (2020). Unpacking mHealth interventions: a systematic review of behavior change techniques used in randomized controlled trials assessing mHealth effectiveness. Digit Health.

[62] Peek ME, Sayad JV, Markwardt R (2008). Fear, fatalism and breast cancer screening in low-income African-American women: the role of clinicians and the health care system. J Gen Intern Med.

[63] Flórez KR, Aguirre AN, Viladrich A, Céspedes A, De La Cruz AA, Abraído-Lanza AF (2009). Fatalism or destiny? A qualitative study and interpretative framework on Dominican women's breast cancer beliefs. J Immigr Minor Health.

[64] Spurlock WR, Cullins LS (2006). Cancer fatalism and breast cancer screening in African American women. ABNF J.

[65] Bondaronek P, Alkhaldi G, Slee A, Hamilton FL, Murray E (2018). Quality of publicly available physical activity apps: review and content analysis. JMIR Mhealth Uhealth.

[66] Brewer LC, Fortuna KL, Jones C, Walker R, Hayes SN, Patten CA, Cooper LA (2020). Back to the future: achieving health equity through health informatics and digital health. JMIR Mhealth Uhealth.

[67] Armaou M, Araviaki E, Musikanski L (2019). eHealth and mHealth interventions for ethnic minority and historically underserved populations in developed countries: an umbrella review. Int J Commun Wellbeing.

