# Supplementary material for: Designing a Cancer Prevention Collaborative Goal-Setting Mobile App for Non-Hispanic Black Primary Care Patients: An Iterative, Qualitative Patient-Led Process
Source: JMIR Form Res. 2022 Mar 24;6(3):e28157. doi: 10.2196/28157 (PMC8990368; doi:10.2196/28157)
Supplement: Multimedia Appendix 1 [file formative_v6i3e28157_app1.docx]

**Appendix 1. Interview Scripts and Early Prototype Examples**

***Interview Script 1***

***Introduction****:*

Hello, I’m ___ and I’m going to walk through some rough designs for a phone app. The app has two purposes: first, to help people commit to making healthy changes that will result in a lower chance of getting cancer, and second, to get support from other people to follow through with their commitment.

I’m going to show you several paper print-outs of our designs and I would like you to react to them as if you were using the app, including tapping on buttons. As we go through each print-out, I’d like you to think out loud and say your reactions as you have them. I don’t want to interrupt your thought process so I’ll stay pretty quiet until you’ve gone through each print-out.

Please remember that we are testing the design and not you, so there are no wrong answers here. 

- Do you have any questions before we get started?

***Task 1: Goal Setup***

**Use page 1-A (Green)**

This first print-out shows what you would see when you use the app for the first time. I will cover the other screens and reveal them as you tap on buttons. Please start here and talk through your thoughts and actions out loud.

- What do you think about the connection between daily habits and cancer?
  - Is this motivating?
  - Should this be highlighted more or less?
- What do you think of these goal categories?
  - If they don’t work for you, what types of goals regarding your habits would you prefer?
- Do you have any other thoughts on these screens before we move on?

***Transition***

Now, you have your goal set up. The next key part of the app is how you’ll report your progress and share it with others.

We are considering two different ways that you can share your progress. I am going to show you a series of screens for each of these two ways.

***Task 2: Check in and share progress with known social ties and/or others***

**Use pages 2-A through 2-D (Pink)**

**[2-A]** These screens show the first way of getting supporters involved. Please take a look and talk through your reaction.

**[2-B]** This series of screens shows how you would report and share your progress.

**[2-C]** This next series of screens shows what would happen when you tap “Everyone” on the last screen.

**[2-D]** Backing up a bit, this screen shows what happened if you tap No when asked if you completed your goal.

- Do you have any other thoughts on these screens before we move on?

***Task 3: Check in and share progress with unknown social ties and/or known friends/family***

**Use pages 3-A through 3-C (Orange)**

**[3-A]** Now we’ll look at our second way of sharing your progress. Let’s back up to the beginning. You have just finished creating your goal, and instead of inviting your friends by their phone number, you’ll see a different screen.

**[3-B]** Next, these screens shows how you’d check in and share your progress this time. The first three screens are the same as what you saw before, but the last one will be different.

**[3-C]** Finally, I have another option for what you could see if you selected No when asked if you met your goal.

- Do you think you would modify your goal? How would you decide to modify it?
- Do you have any other thoughts on these screens before we move on?

**Wrap-Up**

Thank you for helping us test these designs. I have a few final questions before we wrap up.

1. Now that you’ve seen all of the screens, what did you think about the process for setting your goal?
2. We went through 2 different ways you could share your progress with others. Which one did you prefer and why?
3. Which option would make you more likely to achieve your goal?
4. Our first option is designed around getting friends and family to achieve their own goal alongside you. Do you think your friends or family would be willing to participate? How many people would you ask?
5. Our second option asked you to make up a username because your progress is automatically shared with everyone else. What did you think about making a username instead of using your real name?
6. Did you learn something from this app that you didn’t know before?

1. Do you think you’d personally use an app like this?

1. Was there anything that you really liked in any of these screens?

1. Was there anything that you disliked or felt could have been left out?

**Finish**

Those are all of my questions. Thank you again for your help.

**Prototype 1**

**
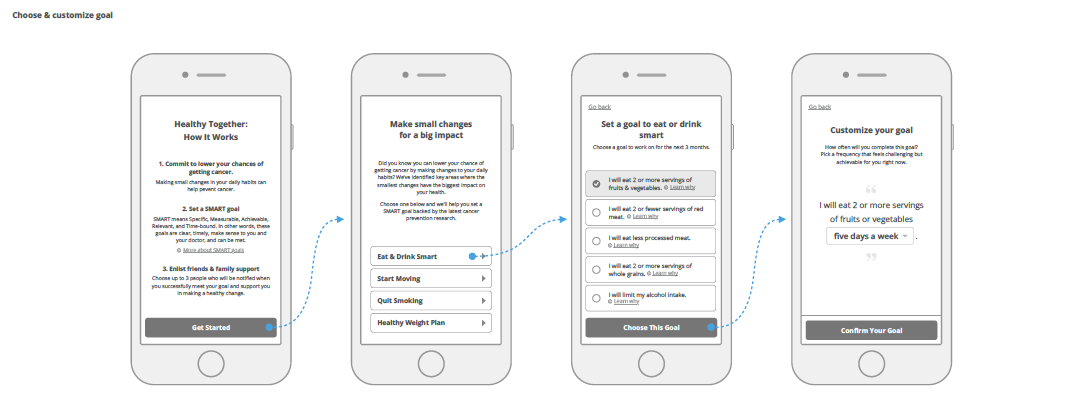
**

**
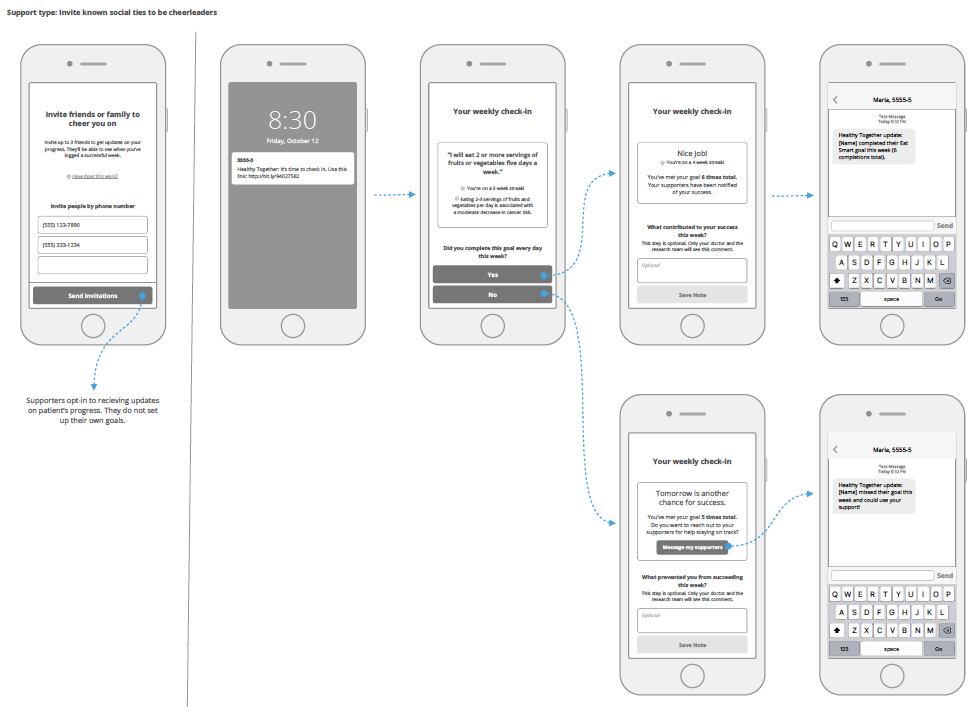
**

**
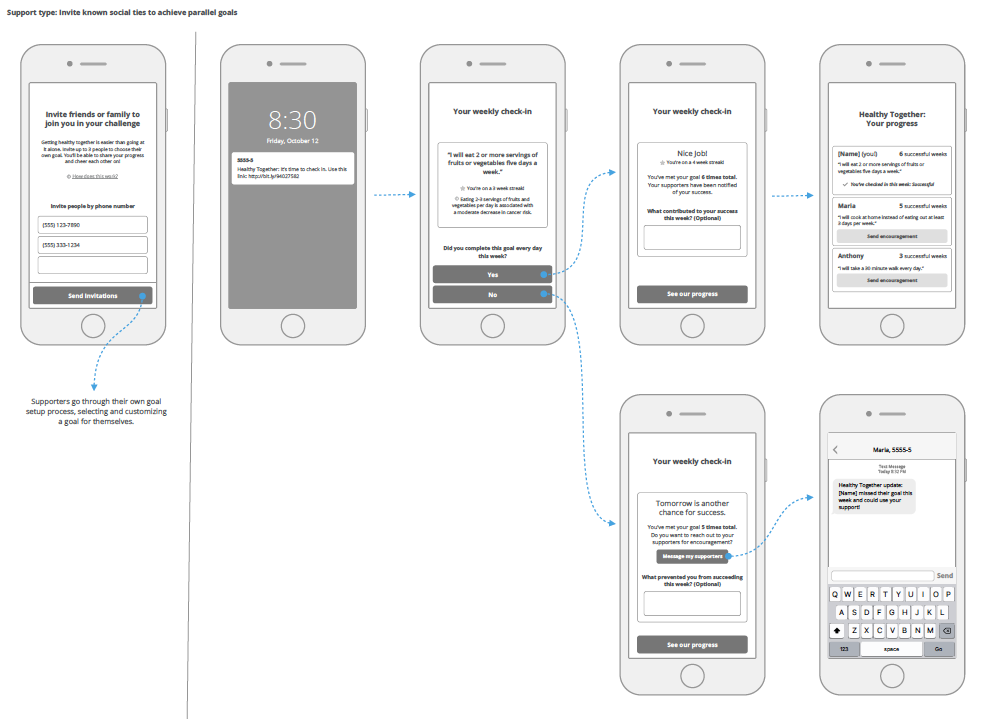
**

**
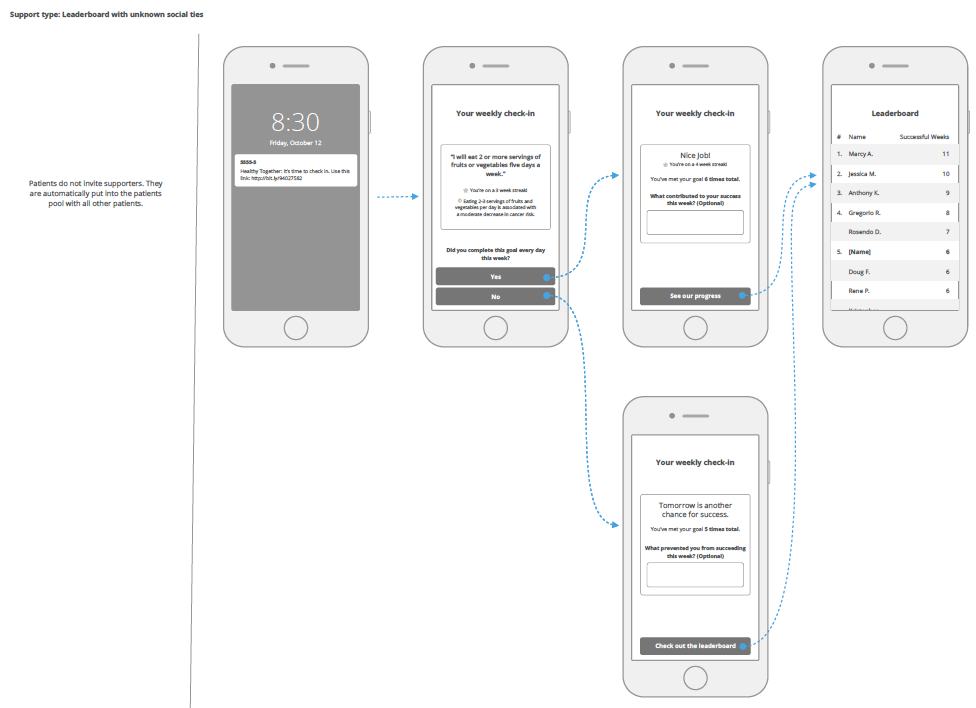
**

***Interview Script 2-modified to reflect end-user testing results from prior week and revised prototype***

***Introduction****:*

I’m going to walk through some rough designs for a phone app. The app has two purposes: first, to help people commit to making healthy changes that will result in a lower chance of getting cancer, and second, to get support from other people to follow through with that commitment.

I’m going to show you several paper print-outs of our designs and I would like you to react to them as if you were using the app, including tapping on buttons. As we go through each print-out, I’d like you to think out loud and say your reactions as you have them. I don’t want to interrupt your thought process so I’ll stay pretty quiet until you’ve gone through each print-out.

Please remember that we are testing the design and not you, so there are no wrong answers.

- Do you have any questions before we get started?

***Task 1: Goal Setup***

**Use page 1-A**

This first print-out shows what you would see when you use the app for the first time. I will cover the other screens and reveal them as you tap on buttons. Please start here and talk through your thoughts and actions out loud.

- What do you think about the connection between daily habits such as diet, exercise, and maintaining a healthy weight and cancer? *(PROMPT: in addition to smoking)*
  - Is this surprising to you?  Did you know this beforehand?
  - Is this motivating?
  - Should this be highlighted more or less?
  - In your opinion, do you think it’s very possible, somewhat possible, or not possible to prevent cancer by making small changes like these?
- What do you think of these goal categories?
  - *(If response is negative)* What types of goals regarding your habits would you prefer?
- Do you have any other thoughts on these screens before we move on?

***Task 2: Set up sharing***

**Use pages 2-A through 2-D**

**[2-A]** Here is what you will see after setting up your goal. **(*Keep right screen covered*)***

- Here you have the option to share with people you know, your friends and family, ***(point to first toggle)*** or people you don’t know who are also working towards cancer prevention ***(point to second toggle)***. You can choose just one of these options, or you can turn both on. What would you turn on?

***IF* participant turns on “Share with friends or family”:**

**[2-B]** After you turn that option on, you will see these screens.

***IF* participant turns on “Share with everyone, anonymously”:**

**[2-C]** After you turn that option on, you will see this screen.

***IF* participant turns on both:**

**[2-B]** You’ll see this screen after turning on the “Share with friends or
 family” option *(point to specified toggle).*

**[2-C]** Then, you’ll see this screen after turning on the “Share with everyone”
 option *(point to specified toggle).*

**[2-A] (**Return to 2-A and reveal right screen*)** After you decide who to share with, you’ll be finished setting up the app.

***Transition***

The next key part of the app is how you’ll report your progress and share it with others. We are considering two different approaches for this part. I am going to show you a series of screens for each of these two ways.

***Task 3: Check in and share – collaborative messaging***

**Use pages 3-A through 3-C**

**[3-A]** These screens shows how you’d check in and share your progress towards your goal. Please take a look and talk through your reaction.

**[3-B]** Before, you had the option to turn on sharing with your friends and family and sharing with people you don’t know who are also working towards cancer prevention. These next screens will show how it would look to share in each of those ways.

- What do you expect to happen when you tap Send Encouragement?

**[3-C]** This shows what would happen if you tap Send Encouragement. In this example, it sends a message individually to one person.

- Would you prefer that this message was sent to a group chat with all your supporters, instead of individually as shown here?
- What would you want this message to say?
- Do you have any other thoughts on these screens before we move on?

***Task 4: Check in and share – competitive messaging***

**Use pages 4-A through 4-C**

**[4-A]** Now we’ll look at our second way of sharing your progress. Just like before, you set up your goal, chose who to share with, and now you’re ready to check in. The first three screens will be the same as what you saw before, but the last one is different.

**[4-B]** Once more, we’ll look at sharing with your friends vs. sharing with others.

**[4-C]** And finally, backing up a bit, this shows what would happen if you didn’t meet your goal ***(refer to check-in screen)*** and tapped “No” when you checked in.

- Do you have any other thoughts on these screens before we move on?

**Wrap-Up**

Now that you’ve seen how the whole app works, I have a few general questions.

1. We went through the process of checking in and sharing your progress two times. The first time, the design encouraged collaboration. The second time, the design encouraged competition. Which one did you prefer and why?

1. This app is designed to get friends and family to achieve goals alongside you. Do you think your friends or family would be willing to participate?
   - How many people would you ask?
2. In this app, we allow you to share your progress with your family and friends, and with a wider pool of people you don’t know. Would you prefer to share only with your friends, only with people you don’t know, or do you like being able to share with both?
   - *[If they saw page 2-C]* What did you think about creating a username?

1. Our app allows you to send messages of encouragement. Would you want the option to chat with everyone in a big group chat, even if you didn’t know them?
2. Did you learn something from this app that you didn’t know before?

1. Do you think you’d personally use an app like this?

1. Was there anything that you really liked in any of these screens?

1. Was there anything that you disliked or felt could have been left out?

- What do you see as the goal of this app?

**
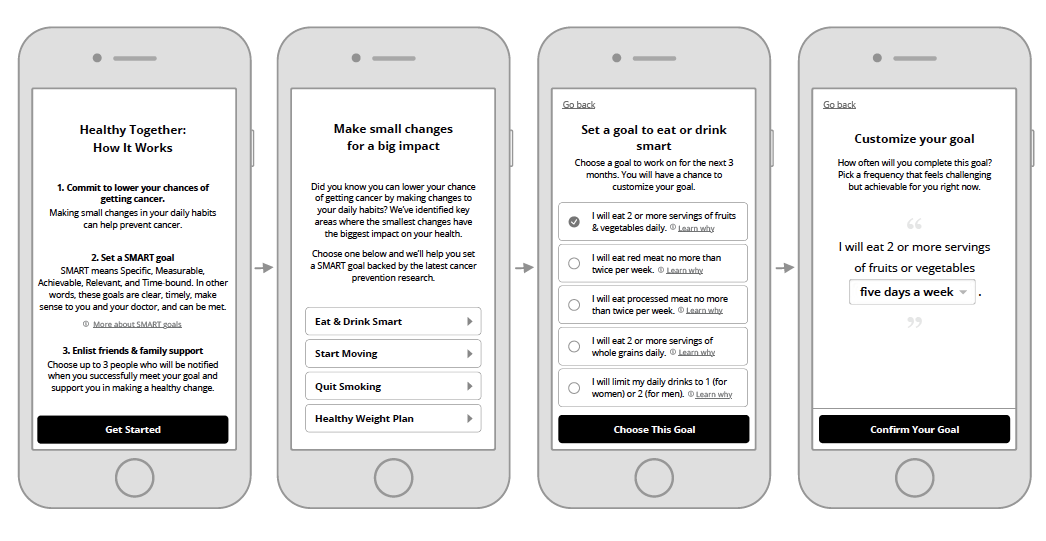
**

**
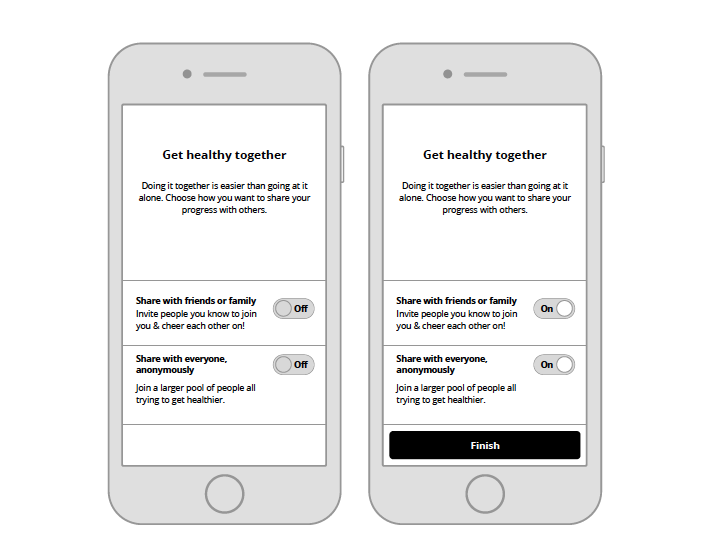
**


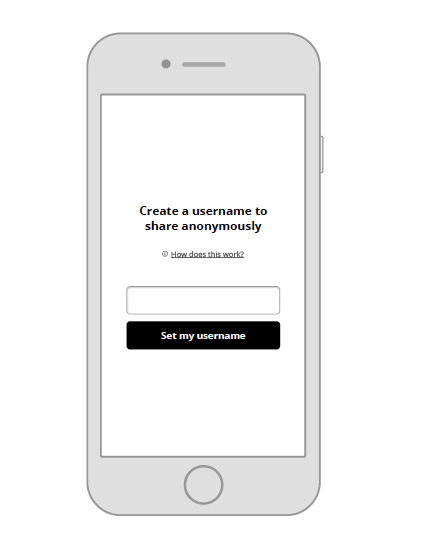
**
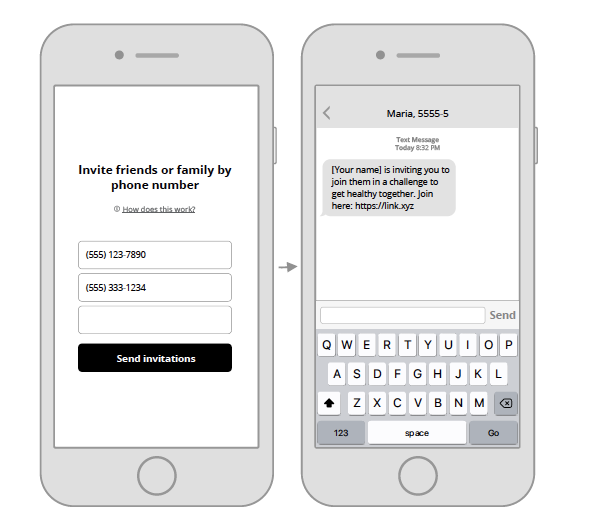
**

**
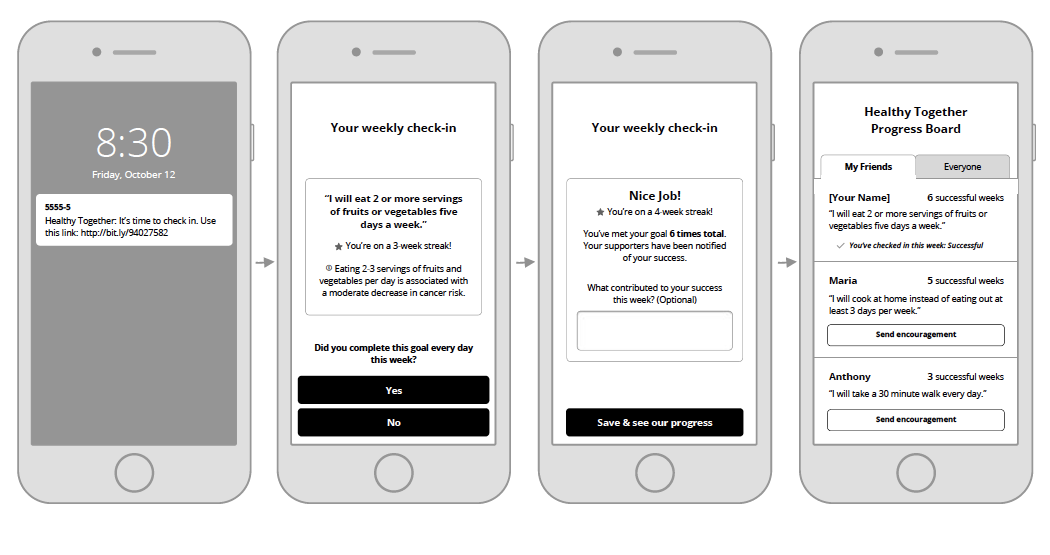
**

**
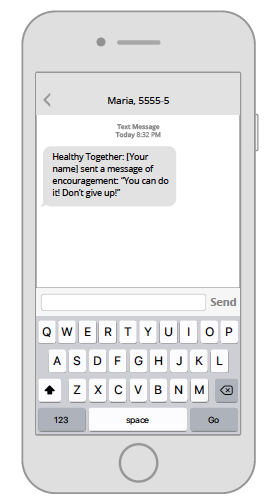

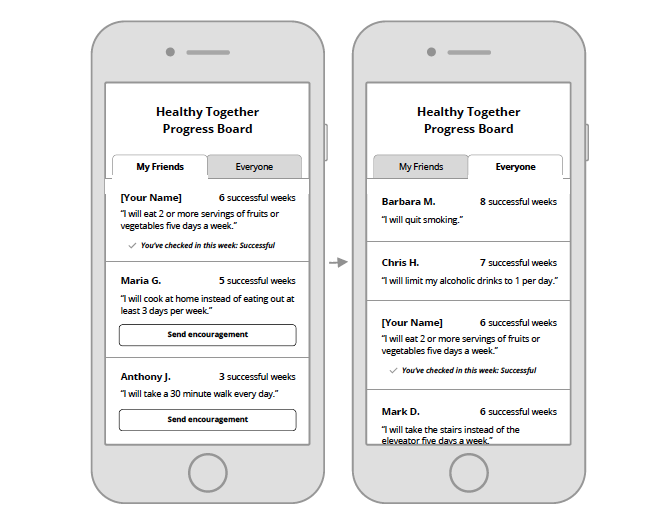
**

**
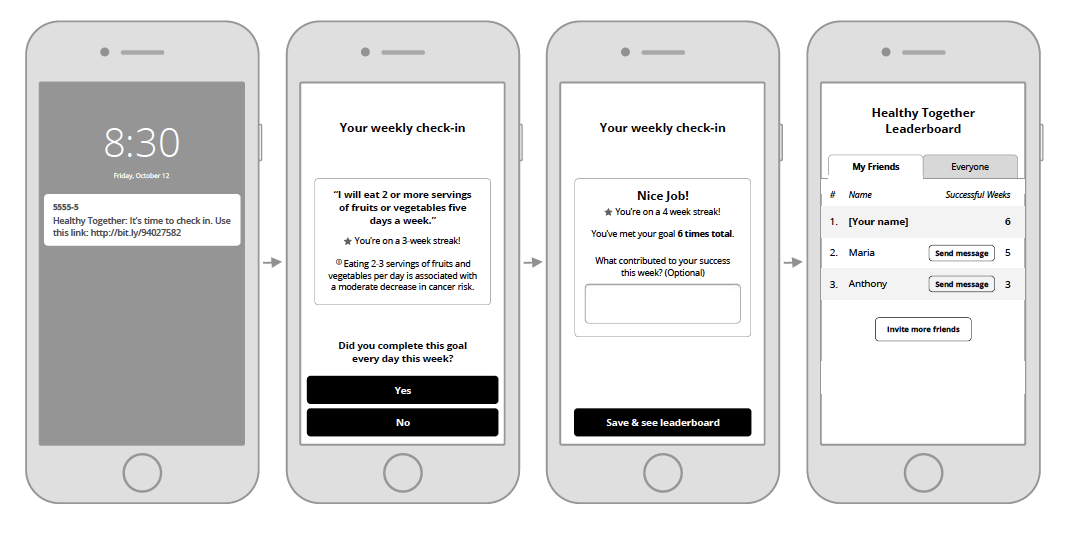
**

**
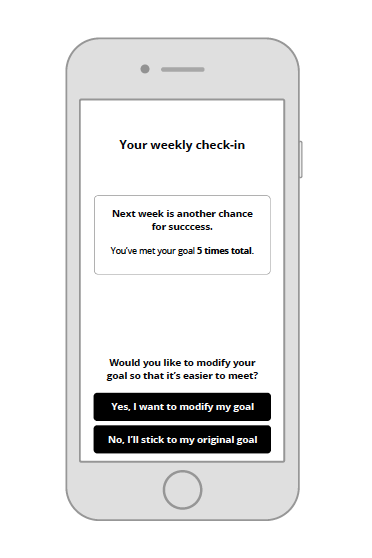

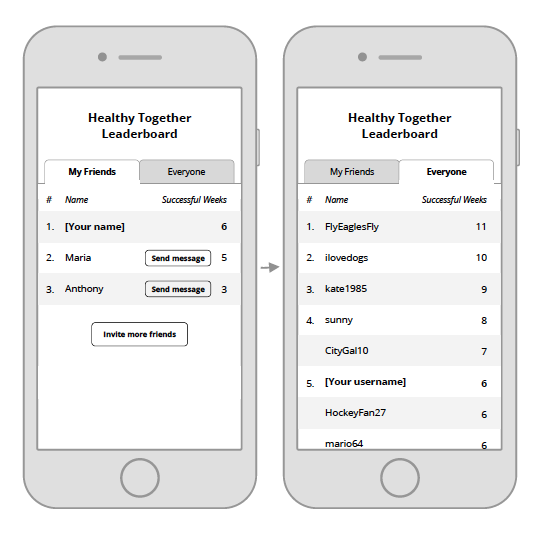
**
