# Supplementary material for: Designing a Cancer Prevention Collaborative Goal-Setting Mobile App for Non-Hispanic Black Primary Care Patients: An Iterative, Qualitative Patient-Led Process
Source: JMIR Form Res. 2022 Mar 24;6(3):e28157. doi: 10.2196/28157 (PMC8990368; doi:10.2196/28157)
Supplement: Multimedia Appendix 2 [file formative_v6i3e28157_app2.docx]

**Appendix 2. Interactive Prototype and Corresponding Interview Guide**

***Link to Prototype:*** [***https://projects.invisionapp.com/share/H6RV5M6MYG3#/screens/361697904_Intro***](https://projects.invisionapp.com/share/H6RV5M6MYG3#/screens/361697904_Intro)

***Interview Guide from initial end-user testing of prototype***

***Introduction****:*

Today we’re enlisting your help in testing the prototype of a phone app. The app has two purposes: first, to help people commit to making healthy changes that will result in a lower chance of getting cancer, and second, to get support from other people to follow through with that commitment.

Using this phone, you’ll use our prototype as if you were using a real app. Because this is just a prototype, not every button is hooked up yet, but the major ones are. I will be able to see what’s happening on the phone through my computer.

As you use the app, say your thoughts and reactions out-loud in real time. I don’t want to interrupt your thought process so I’ll mostly stay quiet until a few key points, when I’ll ask you questions about your experience. Please remember that we are testing the app and not you, so there are no wrong answers. If you’re confused about something, or you see something you like or that you don’t like, that’s all good feedback for us to hear. Do you have any questions before we get started?

- Record goal chosen in row 4
- Record sharing settings chosen in row 7

***After goal and sharing setup:***

- In the beginning we asked you to choose a goal and you chose ____. Do you have any thoughts or reactions about that process?
- Next, we asked you to decide how you want to share: with friends and family, with a larger anonymous group, or both. You chose _____. Do you have any thoughts or reactions about that process?
- Do you have any other thoughts or reactions to any of the screens you just saw, overall?

Now we are going to skip ahead. You have been working on your goal for a few weeks now, and once a week the app checks in with you to see how your progress is going.

For the next portion of the prototype, we’ve set it up as if your goal is eating more fruits and vegetables and you choose to share with both friends and everyone in the larger group.

*In the prototype, move to the screen where there is a text message notification on the lock screen.*

***When they seem to have reached the end, or don’t know what else to do:***

*(If they didn’t navigate to any of the screens below, take them back to those screens:)* Before we do our final survey, I want to show you a few screens that you didn’t get to.

- Friends progress board
- Everyone progress board
- Sharing settings
- Viewing a profile
- Sending encouragement

*Then ask final questions:*

- You went through the process of checking in and recording your progress on your goal. Did you have any thoughts or reactions on that process?
- After that, you saw a screen that listed all of your friends and their progress. Did you have any thoughts or reactions on that?
- You also saw a screen that listed everyone in a larger group and their progress. Did you have any thoughts or reactions on that?
- You went through some screens that showed the process of sending a message. Did you have any thoughts or reactions on that?
- Do you have any other thoughts or reactions about any of the screens you just saw, overall?
